# Recent Advances in Molecular Genetic Tools for *Babesia*

**DOI:** 10.3390/vetsci8100222

**Published:** 2021-10-08

**Authors:** Hassan Hakimi, Masahito Asada, Shin-ichiro Kawazu

**Affiliations:** 1 Department of Disease Control, National Research Center for Protozoan Diseases, Obihiro University of Agriculture and Veterinary Medicine, Hokkaido, Obihiro 080-8555, Japan; skawazu@obihiro.ac.jp; 2 Department of Global Cooperation, National Research Center for Protozoan Diseases, Obihiro University of Agriculture and Veterinary Medicine, Hokkaido, Obihiro 080-8555, Japan; masada@obihiro.ac.jp

**Keywords:** *Babesia*, genome, genetic tools

## Abstract

Development of in vitro culture and completion of genome sequencing of several *Babesia* parasites promoted the efforts to establish transfection systems for these parasites to dissect the gene functions. It has been more than a decade since the establishment of first transfection for *Babesia bovis*, the causative agent of bovine babesiosis. However, the number of genes that were targeted by genetic tools in *Babesia* parasites is limited. This is partially due to the low efficiencies of these methods. The recent adaptation of CRISPR/Cas9 for genome editing of *Babesia bovis* can accelerate the efforts for dissecting this parasite’s genome and extend the knowledge on biological aspects of erythrocytic and tick stages of *Babesia*. Additionally, *glmS* ribozyme as a conditional knockdown system is available that could be used for the characterization of essential genes. The development of high throughput genetic tools is needed to dissect the function of multigene families, targeting several genes in a specific pathway, and finally genome-wide identification of essential genes to find novel drug targets. In this review, we summarized the current tools that are available for *Babesia* and the genes that are being targeted by these tools. This may draw a perspective for the future development of genetic tools and pave the way for the identification of novel drugs or vaccine targets.

## 1. Introduction

**Figure 1:**
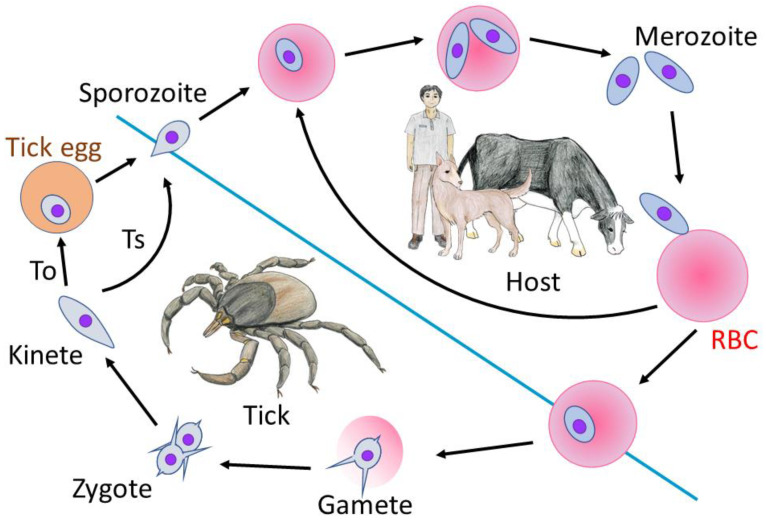
The life cycle of *Babesia* spp. The infection starts when *Babesia* sporozoites are injected into the mammalian host during the blood meal and directly invade and multiply in the RBCs. A subset of the parasite population transforms into gametocytes in the host or upon taken up by the tick where they produce gamete in the tick midgut. Gametes produce diploid zygotes following fertilization. Zygotes invade midgut epithelium and undergo meiotic division which produces kinetes. Kinetes invade and multiply in several organs including salivary glands which results in transstadial transmission (Ts). In most of *Babesia* spp. except *B. microti*, kinetes invade ovaries and eggs which results in parasite transmission into offspring (transovarial transmission, To).

*Babesia* are unicellular, apicomplexan tick-borne parasites that have a great economic impact on the livestock industry, pet animal and wildlife health, and a growing concern of human health due to accidental infections by zoonotic *Babesia*. The parasites were initially discovered at the end 19th century by Babes in cattle with hemoglobinuria [1]. Since then, more than 100 different *Babesia* spp. were found to infect a wide range of mammals and are considered to be the second most common blood parasites after trypanosomes [2,3]. Mammalian hosts are infected by sporozoites during the tick blood meal and *Babesia* parasites exclusively invade and multiply within red blood cells (RBCs) (Figure 1). The sexual stage or gamogony happens in the tick midgut, which is followed by kinetes formation and migration to salivary glands, and ultimately sporozoites production which is called transstadial transmission [4]. These sporozoites can infect the next intermediate host following tick molting. A majority of *Babesia* spp. except *B. microti* have transovarial transmission in which the parasites spread from the mother tick to the offspring [5]. Babesiosis can have varying degrees of severity based on the parasite species, age, and immunological status of the host and coinfection with other pathogens [3]. The clinical features of the disease include fever, anemia, hemoglobinuria, jaundice, and splenomegaly and can cause severe complications and fatality in some virulent species [3]. The control strategies consist of treatment of patients, tick control using acaricide, live attenuated vaccine in case of bovine babesiosis, and soluble parasite antigens-based vaccine for canine babesiosis [6,7]. The emerging resistance to drugs and acaricides and lack of effective vaccines are the main obstacles to controlling babesiosis. 

There is an urgent need to develop new drugs and find vaccine candidates against babesiosis. A better understanding of the biology of *Babesia* spp. facilitates identification and characterization of new vaccine and drug targets and assists to understand the molecular basis of current drug resistance. The advanced progress in functional characterization of related apicomplexan parasites, *Plasmodium* spp. and *Toxoplasma gondii*, can shed light on the conserved genes and pathways in *Babesia*; however, there are numerous unique genes that lack homology in related parasites or model organisms and are *Babesia* specific including several multigene families. Identification of conserved biological pathways across *Babesia* spp. can pave the way for finding pan-*Babesia* drug targets [8]. Several *Babesia* parasites are being adapted to in vitro culture [9,10,11,12,13,14,15], which facilitate high-throughput compound screens to find novel drugs [16]. However, the molecular targets of the currently available drugs are lagging mainly due to limited genetic tools for these parasites. The genome sequence of *Babesia bovis*, the causative agent of bovine babesiosis, was first to be released and followed by several other *Babesia* spp. [17,18,19,20,21]. This advancement motivated researchers to identify gene regulatory regions and further the establishment of genetic modification techniques [22]. Such tools have been used to study parasite biology in the erythrocytic stage and the identification of tick stage-specific proteins [23,24,25,26,27,28]. In this review, we summarized the current tools that are available for genetic modification of *Babesia* spp. and draw the possible road for future advancement in this field. 

## 2. Genome and Genetic Tools for *Babesia*

**Figure 2:**
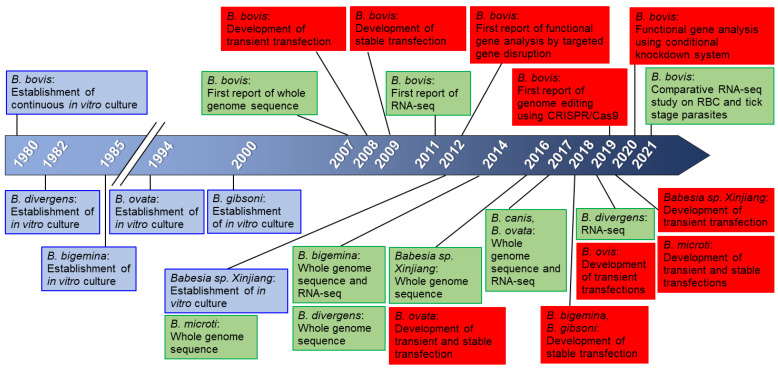
Establishment of in vitro culture, release of genome and transcriptome sequences, and development of genetic tools for *Babesia.* The development of in vitro culture (blue box), release of the whole genome and transcriptome sequences (green box), genetic tools (red box) are shown in chronological order.

Complete genome sequences of *B. bovis*, *B. microti*, *B. bigemina*, *B. divergens*, *Babesia* sp. (*Xinjiang*), *B. canis*, and *B. ovata* are available [17,18,19,20,21,29,30]. A chronological timeline of releasing of *Babesia* genome and developed genetic tools are shown in Figure 2. The transcriptomes of several *Babesia* in normal or modified culture conditions, virulent and attenuated strains, and tick stage of the parasite are available [20,26,30,31,32,33,34]. These transcriptome data could be used to show stage-specific gene expression, transcription start site, identification of alternative splicing, and better annotation of the genome. Greater than half of the genes in the genome of *Babesia* parasites have no predicted function which includes the genus-specific genes and several multigene families [8]. Genetic tools are needed to aid in the functional characterization of these genes. 

Episomal expression of the transgene could be performed by transfection of circular plasmid DNA while genome integration of the transgene through single or double-crossover homologous recombination could be achieved using linear plasmids [35,36]. Transient transfection of *B. bovis* merozoites was reported shortly after the release of this parasite genome [37]. It was followed by two independent reports of stable transfection for this parasite which *blasticidin S deaminase* (*bsd*) and *human dihydrofolate reductase* (*hdhfr*) was used for the selection of transgenic parasites which confer resistance to blasticidin S and WR99210, respectively [35,38]. Given that the commercially available WR99210 from Sigma-Aldrich is an isomer of the original product from Jacobus pharmaceutical and not functional for the selection of transgenic parasites expressing hDHFR [39], application of *hdhfr*/WR99210 is limited to the laboratories that have access to the latter product. However, *hdhfr* also confers resistance to pyrimethamine and this drug could be used instead of WR99210. These advancements in genetic manipulation of *Babesia bovis* genome inspired other scientists to establish transfection tools for several other *Babesia* spp. Transient transfections for validation of promoter activity were established for *B. bovis*, *B. bigemina*, *B. ovata*, *B. gibsoni*, *B. ovis*, *B. microti* and *Babesia* sp. *(Xinjiang)* [15,37,40,41,42,43,44]. Additionally, stable transfections were reported for several *Babesia* parasites including *B. ovata*, *B. gibsoni*, *B. bigemina*, and *B. microti* [41,45,46,47]. As for most of these parasites, the availability of a robust in vitro culture system was a prerequisite to establishing the genetic tools. Regarding *B. microti*, although stable transfection has been reported in the in vivo condition, transgenic parasites were enriched using fluorescent-activated cell sorting [47]. It is needed to optimize drugs for the selection of transgenic parasites in future studies.

Given the economic importance, availability of genome and several transcriptome data, and availability of several phenotype assays, main progress on *Babesia* biology in the tick and the mammalian host has been accomplished using *B. bovis*. However, of ~3800 genes in the genome of *B. bovis*, only 13 genes have been targeted for epitope tagging, producing point mutation, or gene disruption (Table 1). Several factors hampered the progress on the application of genetic tools for *Babesia* spp., such as the low efficiency of the transfection system and limited selectable markers. Bio-Rad electroporation device was initially used to transfer plasmid DNA to parasite nucleus [38] and transfection efficiency was improved using Amaxa nucleofector device [35]. Currently, two selectable markers are available for *Babesia* parasite, *bsd* and *hdhfr* [35,38]. Thus, sequential genetic manipulation or complementation studies are possible. Asada et al. (2015) used both selection systems for studying *tpx-1* gene knockout and complementation study in *B. bovis* [24]. Future application of negative selection brings the possibility of recycling the selection markers to perform sequential gene knockout. Of the targeted genes in *B. bovis*, *elongation factor 1-alpha* (*ef1-α*), *thioredoxin peroxidase-1* (*tpx-1*), *rad51* and several tick-stage genes such as *hap2*, *6-Cys E*, and *6-Cys A* and *B* were shown to be dispensable in the erythrocytic stage of the parasite [27,28,35,38,48,49]. These gene loci could be used for knock-in or insertion of a fluorescent reporter gene such as *gfp* for imaging studies. Genetic tools have been used for endogenous or episomal tagging of genes to confirm the localization of their product [25,26]. To dissect segmental gene conversion through homologous recombination in *B. bovis*, Mack et al., (2019 2020) disrupted *rad51* gene and showed that it is not essential for parasite growth in vitro. However, these parasites lost homologous recombination-dependent gene integration and showed a reduction of in-situ transcriptional switching [9,48]. Recently, a novel multigene family encoding protein with multi-transmembrane domain (*mtm*) was discovered and overexpressing studies showed that their expression was linked to blasticidin S resistance [26]. Two proteins, SBP2 truncated copy 11 and BbVEAP, were shown to affect cytoadhesion of iRBCs to endothelial cells, thus are involved in *B. bovis* virulence [26,50]. Upregulation of SBP2 truncated copy 11 reduced binding of iRBCs to endothelial cells, while knockdown of BbVEAP, VESA1-export associated protein, decreased ridge numbers and abrogated cytoadhesion of iRBCs [26,50]. BbVEAP is the first piroplasm-specific protein shown to be essential for parasite development in the RBC [26]. Perforin like protein 1 (Plp1) was shown to be important for parasite egress where knockout parasites had a growth defect with the appearance of RBCs infected with multiple *B. bovis* [51]. All these studies have been done in the erythrocytic stage of the parasite, and so far, no conditional tools are available for dissecting gene functions in the tick stage. Establishment of genetic tools and characterization of tick stage-specific promoters can accelerate the identification of genes important for the tick stage and assist in finding novel targets for transmission-blocking vaccines.

## 3. Genome Editing Using CRISPR/Cas9

**Figure 3:**
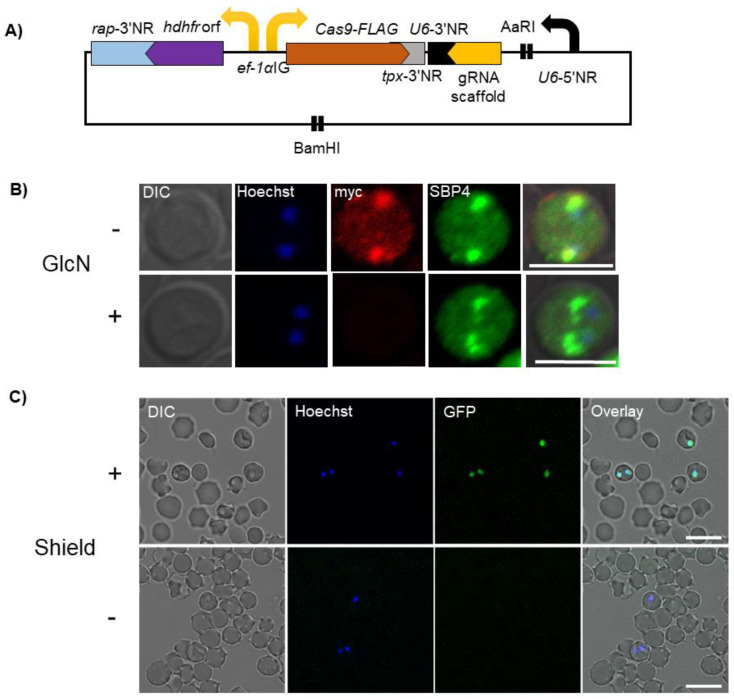
Schematic of CRISPR/Cas9 plasmid and conditional systems to regulate gene expression for *Babesia bovis*. (**A**) Cas9 and *hdhfr* are driven by *ef1-α* bidirectional promoter or intergenic region (IG) while *U6 spliceosomal RNA* promoter or 5′ noncoding region (5′-NR) drives gRNA expression. The gRNA and donor DNA are inserted into AaRI and BamHI sites in the plasmid, respectively. (**B**) Indirect immunofluorescence microscopy test of BbVEAP-myc-*glmS* parasite in the presence (+) or absence (−) of glucosamine, GlcN (α-myc, red and α-SBP4 (control), green). The parasite nuclei were stained with Hoechst 33,342 (Hoechst, blue). Scale bar = 5 μm. (**C**) Live fluorescence microscopy images of green fluorescent protein- destabilizing domain (GFP-DD)-expressing parasites in the presence (+) or absence (−) of Shield. The parasite nuclei were stained with Hoechst 33,342 (Hoechst, blue). Scale bar = 10 μm. DIC, differential interference contrast.

Site-specific nucleases include zinc-finger nuclease (ZFN), transcription activator-like effector nucleases (TALEN), and Clustered regularly interspaced short palindromic repeat (CRISPR)/Cas9 that selectively produce a double-strand break at a defined genomic site. CRISPR/Cas9 is an acquired immune response in prokaryotes to protect them against invading bacteriophages [52]. This system had successfully been repurposed for genome editing of several organisms, accelerating and revolutionizing their functional genomics. The double-strand break produced by CRISPR/Cas9 should be repaired, which in many organisms happens through error-prone non-homologous end joining (NHEJ), and produces indels, subsequently disrupting the gene function [53]. *Babesia* spp. lack NHEJ and need template DNA for the repair of double-strand break of DNA [8]. CRISPR/Cas9 system had been adapted to *B. bovis* and was shown to be efficient for gene editing purposes such as epitope tagging, the introduction of point mutation, and production of gene knockout [25,26]. As shown in Figure 3A, a single plasmid was used to express Cas9, gRNA and the donor template DNA [25]. The *ef1-α* bidirectional promoter simultaneously drives the expression of Cas9 and hDHFR, whereas *U6 spliceosomal RNA* promoter was used to drive gRNA. gRNA and ~1 kb donor DNA as a template designed based on the target gene could be inserted into AaRI and BamHI sites in the plasmid, respectively. While this single plasmid transfection system was efficient for gene editing, the authors found the integration of the plasmid into the genome which necessitates the application of negative selection to remove the plasmid backbone and recycle hDHFR for sequential gene editing [25]. Integration of CRISPR plasmid into genome tends to happen when a single plasmid is being used in the rodent malaria parasite, *Plasmodium yoelii* [54]. Expression of two gRNAs using a novel ribosome-mediated CRISPR system or genome integration of Cas9 together with using linear donor DNA prevents integration of plasmid and allows recycling of drug selection cassette [55,56]. Application of inducible Cas9 and stage-specific expressed Cas9 can enhance our ability to dissect *Babesia* genome during the erythrocytic or tick stage. Null Cas9 could be employed to precisely guide the epigenetic regulators to the transcription start site to control transcription of the target gene [57]. Recently, a new class of CRISPR/Cas system, Cas13, was identified which targets RNA. Cas13 has been validated for transcriptome engineering such as RNA editing, RNA knockdown, and manipulating RNA splicing [58]. It was shown that Cas13 has favorable efficiencies in mammalian and plant cells with no off-target, unintended knockdown of genes, unlike the RNA interference (RNAi) system making it a promising high-throughput genetic tool for *Babesia*. 

## 4. Conditional Knockdown Systems

Given that *Babesia* genome is haploid in erythrocytic and most of the developmental stages in the tick, conventional knockout systems are not suitable to be used for functional characterization of essential genes. Therefore, conditional systems are needed to dissect the functions of indispensable genes to gain insights into druggable targets. Conditional or inducible expression systems can regulate target expression at the genome, transcriptome, or protein level. There is a single report describing the conditional knockdown of mRNA in *B. bovis* using self-cleaving ribozyme [26]. The *glmS* ribozyme from Gram-positive bacteria [59] could be activated by glucosamine-6-phosphate. The knockdown of BbVEAP in the presence of inducer was ~90% at protein level (Figure 3B) [26]. This reduction confirmed the role of BbVEAP in parasite development in the RBC, VESA1 export and cytoadhesion of iRBCs to endothelial cells [26,60]. Riboswitch system could be simply employed by insertion of *glmS* sequence at 3’ non-coding region of the gene of interest open reading frame, downstream of the stop codon and is a promising method to be used for mRNA knockdown of *Babesia*. RNAi has been used in one study to evaluate the effects of several genes in *B. bovis* growth in the culture [61]. Because *Babesia* parasites lack RNAi machinery [8], the applicability of this system requires further validation. 

To study the protein function, protein level could be manipulated by inducing premature degradation by fusing protein to destabilizing domain or translocation of the target protein by a method called knock sideways, KS [62,63]. The advantage of targeting the protein of interest is the fast action of this system that could be leveraged for studying the rapid biological process [64]. We have validated FK506-binding protein (FKBP)-based destabilizing domain (DD) by fusing GFP with DD (Figure 3C, unpublished data). FKBP-DD could be fused to the N- or C-terminus of the target protein. DD could be stabilized by the addition of Shield 1 and in the absence of Shield 1, the target protein degradation will be promoted via the proteasome. The applicability of the DD system for *Babesia* requires further investigation; however, this system is not suitable for the membrane or secreted proteins that are not accessible to the proteasome in the parasite cytoplasm [64,65]. KS which initially called anchor-away is based on conditional tethering of the protein of interest by rapamycin-dependent dimerization where target protein is fused with FKBP and additionally, FRB is fused to a protein with different cellular localization called mislocalizer [63,66]. The addition of rapamycin results in the relocation of the target protein preventing its function. KS requires prior information regarding localization of target protein, but was shown to be an efficient method to study protein function in several organisms including *Plasmodium* [66,67]. Thus, it could be the method of choice for studying protein function in *Babesia*. 

Conditional knockout methods for genes are not developed for *Babesia*. Conditional deletion of a target gene using dimerizable Cre recombinase (DiCre) has been established for *Plasmodium* spp. and was shown to be efficient for several targets in the in vitro and in vivo models [64,65,68]. This system has two compartments—DiCre, in which its inactive two proteins are fused upon rapamycin addition, and a short targeting sequence called *loxP.* The *loxP* sequences are inserted upstream and downstream of the locus, which is targeted by DiCre and excises the locus. Additionally, a split Cas9 that becomes functional after dimerization could be used for conditional induction of double-strand break for genome editing [69]. 

## 5. In Vitro Culture of *Babesia* and Transfection

In recent years, there have been major technical advances to genetically manipulate *Babesia*. The main prerequisite for the establishment of transfection systems for the majority of these parasites is the availability of in vitro culture. Although several *Babesia* species could be cultured in vitro, these methods are not all well optimized. The primary concern for continuous culture of these parasites is the need for animal serum, cryopreservation methods, and continuous supplement of fresh host erythrocytes. To overcome these challenges, several groups established the application of serum-free mediums, GIT, for *B. bovis* [70], *B. bigemina* and *B. divergens* [71], or replaced animal serum with high-density lipoprotein [72], or Albumax I [73] for *B. divergens*, or lipid mixture for *B. bigemina* [74]. While it is known that different batches of host serum have content variations and can modify drug effects on *Babesia* [70], how these methods affecting transfection efficiencies are unclear. However, standardization of in vitro culture and cryopreservation methods using commercially available products [75] can assure reproducible transfection efficiencies and recoveries of cryopreserved stocks across different laboratories. Current *B.* *microti* transfection is based on in vivo experiments and it is hard to maintain this parasite in vitro [76]. Short term in vitro selection was shown to be efficient for generating transgenic rodent malaria parasite, *P. berghei* [77]. Optimization of in vitro culture for this parasite can open up the possibility of application of currently used drugs in the in vitro culture system, WR99210 and blasticidin S, for selection of transgenic *B. microti*.

## 6. Future Perspective

The slow progress in functional characterization of *Babesia* genes is partially due to the low efficiencies of transfection for these parasites, which needs further improvement. Currently, iRBCs are being used for transfection. Large-scale preparation of parasite merozoites is available and may improve the efficiency of transfection [60]. 

Limited conditional tools are available to study essential genes in *Babesia*. Conditional tools that can modulate gene expression at the genome, transcriptome, or protein levels are needed. Additionally, non-homologous end joining (NHEJ) does not exist in piroplasms; thus, high-throughput screening methods such as CRISPR/Cas9 mediated gene disruption [78] are not applicable for these parasites. The development of high-throughput tools such as CRISPR/Cas13 for *Babesia* will pave the way for functional characterization of multigene families and genome-wide functional characterization to identify the essential genes and pathways to prioritize research for drug discovery. 

## Figures and Tables

**Table 1:** List of *B. bovis* genes targeted for gene disruption, tagging, or overexpression.

Gene Product	Gene ID	Targeted Method	Phenotye	Reference
Elongation factor 1-alpha (ef1-α)	BBOV_IV010620	Knockout	Not essential for in vitro growth	[23,35,36,38]
Thioredoxin perxidase 1 (Tpx-1)	BBOV_II004970	Knockout	Not essential for in vitro growth, increased sensitivity to nitrosative stress	[24,25]
Hap2	BBOV_III006770	Knockout	Not essential for in vitro growth	[28]
6-Cys *E*	BBOV_II006640	Knockout	Not essential for in vitro growth	[27]
6-Cys A and B	BBOV_II006600, BBOV_II006610	Double knockout	Not essential for in vitro growth	[49]
Thioredoxin perxidase 1 (Tpx-1)	BBOV_II004970	Point mutation	Not essential for in vitro growth, increased sensitivity to nitrosative stress	[25]
Spherical Body Protein 2 (SBP2) truncated copy 11	BBOV_III006540	Knockin into *ef1-α* locus	Reduction in binding of iRBCs to endothelial cells	[50]
Spherical Body Protein 3 (SBP3)	BBOV_I004210	Epitope tagging	Protein localization was confirmed with epitope tagging.	[25]
Rad51	BBOV_II003540	Knockout	Not essential for in vitro growth, increased sensitivity to methylmethane sulfonate, loss of HR-dependent integration, and reduction of in situ transcriptional switching	[9,48]
Multi-transmembrane protein (mtm)	BBOV_III000010, BBOV_III000060	Episomal overexpression	Reverting blasticidin S resistance	[26]
VESA1-export associated protein (Bbveap)	BBOV_III004280	Knockdown	Slow growth, abrogation of cytoadhesion	[26]
Perforin like protein 1 (Plp1)	BBOV_IV001370	Knockout	Lower growth rate in vitro	[51]

## Data Availability

Not applicable.
